# Multi-omics integration and experimental validation reveal the mechanism of berberine against triple-negative breast cancer

**DOI:** 10.3389/fphar.2026.1811985

**Published:** 2026-05-28

**Authors:** Ying Zhang, Tao Lu, Kaixuan Liu, Bangmei Zhang, Shu Xu

**Affiliations:** 1 Department of Pathology, Guizhou Medical University, Guiyang, China; 2 Department of Pathology, Affiliated Hospital of Guizhou Medical University, Guiyang, China; 3 Pathology Morphology and Molecular Laboratory of the Affiliated Hospital of Guizhou Medical University, Guiyang, China

**Keywords:** berberine, molecular docking, network pharmacology, single-cell sequencing, triple-negative breast cancer

## Abstract

**Introduction:**

Triple-negative breast cancer (TNBC) is an aggressive subtype of breast cancer lacking effective therapeutic options. Berberine (BBR), a natural isoquinoline alkaloid, has shown anti-tumor potential, but its systemic mechanism of action in TNBC has not been fully elucidated.

**Methods:**

This study adopted an integrated strategy combining network pharmacology, transcriptomic analysis, molecular docking, molecular dynamics simulation, and multi-level *in vitro* experimental validation. Targets of BBR were predicted through multiple databases, TNBC-related genes were screened by integrating GEO and TCGA data, key targets and tumor microenvironment characteristics were analyzed using WGCNA, PPI networks, and single-cell sequencing data, and the biological effects of BBR were validated through molecular docking, dynamics simulation, and cellular experiments.

**Results:**

A total of 182 common targets of BBR and TNBC were identified, with 12 genes, including SRC, STAT3, and EGFR, being identified as hub targets. Immune infiltration analysis indicated that BBR targets were associated with the tumor immune microenvironment. Single-cell sequencing showed that these targets are primarily enriched in tumor cells and macrophages, and molecular docking and dynamics simulations revealed that BBR has high affinity with SRC (−8.8 kcal/mol). *In vitro* experiments confirmed that BBR can concentration-dependently inhibit TNBC cell proliferation, induce apoptosis, increase ROS levels, and significantly reduce the proportion of CD133^+^ stem cells and tumor sphere formation capacity. Western blot analysis showed that BBR downregulated the activity of the p-PI3K/p-AKT and p-SRC/p-STAT3 pathways.

**Conclusion:**

BBR inhibits TNBC cell proliferation, induces apoptosis, weakens tumor stemness, and may regulate the tumor immune microenvironment through multi-target, multi-pathway synergistic effects. This study systematically reveals the integrative mechanism of BBR against TNBC, providing a theoretical basis for its development as a multi-target natural anti-tumor drug.

## 1 Introduction

Breast cancer is one of the most common malignant tumors among women worldwide and is also the second leading cause of cancer-related deaths (Giaquinto et al., 2022). TNBC is a distinct subtype of breast cancer characterized by the absence of estrogen receptor (ER) and progesterone receptor (PR), as well as the lack of HER2 amplification, accounting for approximately 10%–15% of all breast cancer cases (Siegel et al., 2019). Compared with other subtypes, TNBC cells exhibit stronger invasive ability and tumor stem cell-like characteristics, with significantly higher risks of recurrence and death (Duffy et al., 2012). Clinically, due to the lack of available endocrine or HER2-targeted therapies, treatment options for patients are limited. Chemotherapy remains the primary treatment, but its efficacy is limited and accompanied by significant toxic side effects (Waks and Winer, 2019). These factors make the exploration of clear and actionable therapeutic strategies a key focus of research.

BBR is a natural isoquinoline alkaloid derived from traditional Chinese medicinal herbs such as Coptis chinensis, with moderate oral bioavailability and the ability to modulate multiple cellular signaling pathways (Meng et al., 2018; Wang et al., 2019). Previous studies have demonstrated that BBR can regulate inflammatory responses and oxidative stress, inhibit tumor cell proliferation, and promote apoptosis (Dian et al., 2012; Sun et al., 2014; Zhou et al., 2019; Asbaghi et al., 2020; Han et al., 2020). In breast cancer models, BBR has been shown to reduce cellular proliferative capacity, inhibit migration and invasion, and attenuate, to a certain extent, characteristics associated with tumor stem cells (Cheng et al., 2015; Li et al., 2018; Rauf et al., 2021). Given the absence of stable and well-established therapeutic targets in TNBC, the multi-pathway modulatory capacity of BBR provides a scientific rationale for its potential application in TNBC.

Recent studies have begun to explore the therapeutic effects of BBR specifically in TNBC. For instance, BBR has been reported to inhibit TNBC cell proliferation and metastasis through pathways such as AMPK/mTOR and to enhance sensitivity to chemotherapeutic agents (Faysal et al., 2025; Li et al., 2026). Despite these encouraging findings, previous investigations have predominantly focused on isolated signaling cascades or single targets. The systematic pharmacological mechanism of action of BBR in TNBC, particularly its potential regulatory effects on the complex tumor immune microenvironment (TME) and cellular heterogeneity has not been fully elucidated. Therefore, the overall coherence among different research findings remains unclear, which limits a deeper understanding of its comprehensive anti-TNBC mechanisms. Although previous studies have revealed multiple effects of BBR in breast cancer, its molecular mechanisms in TNBC remain unsystematic, and key targets and signaling pathways lack comprehensive integration.The relationships and overall coherence among different research findings remain unclear, which limits a deeper understanding of its anti-TNBC mechanisms.

Based on integrated bioinformatics analyses, this study further combined molecular-level functional assessment with cellular experimental validation to systematically investigate the potential targets and key signaling pathways of BBR in TNBC, and to analyze its regulatory effects on TNBC cell proliferation, apoptosis, oxidative stress, and the maintenance of tumor stemness. The findings contribute to elucidating the anti-tumor mechanisms of BBR from a multi-level perspective and provide a theoretical basis for the application of natural products in TNBC treatment and drug development.

## 2 Materials and methods

### 2.1 Reagents and materials

The HCC1937 and MDA-MB-231cell lines were obtained from the Cell Bank of the Chinese Academy of Sciences. The primary experimental reagent was BBR, and reagents used for cell proliferation and apoptosis assays included the Cell Counting Kit-8 (CCK-8), EdU Cell Proliferation Assay Kit, Annexin V-APC/7-AAD Apoptosis Detection Kit. Primary antibodies used for Western blot and related immunodetection included GAPDH, BAX, BCL-2, Cleaved Caspase-3, AKT, p-AKT, PI3K, p-PI3K, SRC, p-SRC, STAT3, p-STAT3, ALDH1A, SOX2 and CD133. Detailed information regarding the sources and usage of all reagents is provided in Tables 1, 2.

**TABLE 1:** Main reagents used in this study.

Reagents	Manufacturer	Cat. No.
DMEM	GBICO	C11995500BT
RPMI1640	GBICO	C11875500BT
ECL	NCM Biotech	P10300
Western BlotPrimary antibody Diluent	Wuhan boster biological technology co, Ltd.	20F27C17
Rapid electrophoresis solution and transfer solution	Wuhan servicebio technology co, Ltd.	G2081-15
Crystal violet	Beijing Solarbio science and technology co.,Ltd.	G1063
Apoptosis kit	MULITISCIENCES	AT105
CCK-8	Wuhan servicebio technology co, Ltd.	BMU106
PBS	BI/VivaCell	C3580-0500
DMSO	Beijing Solarbio science and technology co., Ltd.	D8371
TBST	Wuhan servicebio technology co, Ltd.	G0004-1L
Gel rapid preparation kit	Wuhan servicebio technology co, Ltd.	PN3011
4% paraformaldehyde	Wuhan servicebio technology co, Ltd.	BL539A
FBS	Vincent Biotechnology (Nanjing) co, Ltd.	086–150
EDU	APEXBIO (United States)	K1078
MammoCultim BasalMedium (Human)	STEMCELL	05,621
ROS assay kit	Shanghai beyotime technology co, Ltd.	S0033S
CD133,PE	ThermoFisher scientific	12–1,338-42
Berberine	Beijing solarbio science and technology co.,Ltd.	B8500

**TABLE 2:** Primary antibodies used in this study.

Antibodies	Cat. No.	Species	Companies	Dulution
BAX	2,772	Rabbit	CST	1:1,000
Bcl-2	15,071	Mouse	CST	1:1,000
AKT	88,800	Rabbit	CST	1:1,000
p-AKT	ET1607-73	Rabbit	Huaan Biotechnology	1:5,000
STAT3	9,139	Mouse	CST	1:1,000
p-STAT3	9,145	Rabbit	CST	1:2000
Cleaved Caspase-3	F0135	Rabbit	Selleck	1:1,000
SRC	F0262	Rabbit	Selleck	1:1,000
p-SRC	F0304	Rabbit	Selleck	1:1,000
PI3K	R22768	Rabbit	Zenbio	1:1,000
p-PI3K	341,468	Rabbit	Zenbio	1:5,000
ALDH1A	MA5-29023	Mouse	ThermoFisher	1:2000
SOX2	11064-1-AP	Rabbit	Proteintech	1:800
GAPDH	GB5004	Rabbit	Servicebio	1:5,000

### 2.2 Prediction of BBR targets

The SMILES string and two-dimensional molecular structure (SDF format) of BBR were obtained from the PubChem database. Based on the aforementioned chemical structure information, potential targets of BBR were predicted using online platforms including PharmMapper, SwissTargetPrediction, and TargetNet. Prior to result integration, predicted targets were filtered according to the scoring criteria of each platform: targets with a prediction probability ≥0.1 in SwissTargetPrediction, a Fit Score ≥0.4 in PharmMapper, and a prediction probability >0 in TargetNet were retained. The threshold of >0 in TargetNet was specifically chosen to capture all potential ligand-target interactions predicted by the model, thereby maximizing the sensitivity of our initial search and minimizing the risk of missing unconventional but relevant pharmacological targets (Yao et al., 2016). Any potential noise or false positives from this inclusive initial screening were subsequently eliminated through a multi-step validation process, including intersection with disease-specific targets and rigorous topological filtering in the PPI network.

### 2.3 Differential gene screening

To ensure the integrity and reliability of the research data, analyses were conducted based on the Gene Expression Omnibus (GEO) and The Cancer Genome Atlas (TCGA) databases. Three TNBC-related datasets were retrieved from the GEO database: GSE31488 (17 TNBC samples and four normal control samples), GSE45827 (41 primary TNBC tissues and 11 normal breast tissues), and GSE65194 (55 primary TNBC tissues and 11 normal breast tissues). After merging the gene expression matrices of the aforementioned datasets, the ComBat function from the sva package in R software was used to eliminate batch effects. To evaluate the effectiveness of this correction and visualize sample distribution, an unsupervised principal component analysis (PCA) was performed. Subsequently, the limma package was employed to screen for differentially expressed genes (DEGs), with the screening criteria set as *P* < 0.05 and |log_2_ fold change| > 2. The TCGA dataset (117 TNBC samples and 11 normal control samples) was used to validate the expression levels of the key differentially expressed genes obtained from the screening, and to assess their potential clinical predictive value.

### 2.4 Weighted gene co-expression network analysis (WGCNA)

Sample quality assessment was performed on the merged transcriptomic data, and outlier samples were identified and removed through hierarchical clustering. Subsequently, the pickSoftThreshold function in the WGCNA package was used to evaluate the network soft-thresholding power. Under the condition that the network satisfied the scale-free topology criterion (topology fitting index *R* ^*2*^ > 0.85), the soft-thresholding parameter was determined for network construction (Langfelder and Horvath, 2008). Based on the selected parameter, the topological overlap matrix (TOM) was calculated, and co-expression modules were identified using the dynamic tree-cut algorithm. The minimum number of genes per module was set to 60, and the module merging threshold was set to 0.25. By calculating the Pearson correlation coefficient between module eigengenes (ME) and the TNBC phenotype, modules with |correlation| > 0.5 and *P* < 0.05 were selected as candidate modules.Within the candidate modules, hub genes were further screened based on module membership (*k*ME>0.8), and their module membership (MM) and gene significance (GS) values were calculated.

### 2.5 Integrated identification of TNBC-associated genes

To identify high-confidence genes associated with the TNBC phenotype, a multi-step integration strategy was employed. First, the intersection between the candidate module genes identified by WGCNA and the differentially expressed genes was obtained to generate a set of initial feature genes. Subsequently, Least Absolute Shrinkage and Selection Operator (LASSO) regression analysis was performed using the glmnet package in R to reduce multicollinearity and select the most discriminative feature genes. Specifically, the cv. glmnet function was utilized with the nfolds parameter set to 10 to perform rigorous 10-fold cross-validation. To avoid overfitting, the optimal penalty parameter λ was determined based on the minimum partial likelihood deviance (lambda.min). Genes with non-zero regression coefficients at this optimal λ were retained as core feature genes.

Concurrently, known TNBC-related targets were systematically retrieved from the GeneCards, OMIM, and Therapeutic Target Database (TTD) using “Triple-negative breast cancer” as the keyword. For GeneCards entries, only genes with relevance scores above the median were retained to ensure strong disease association. Finally, the feature genes identified via LASSO regression were integrated with the database-derived genes, and duplicate entries were removed to establish a comprehensive set of candidate disease targets for subsequent network pharmacology analysis.

### 2.6 Screening of therapeutic targets and PPI analysis

To systematically integrate different data modalities, the raw predicted protein targets of BBR from the pharmacological databases were first standardized and mapped to their official Gene Symbols using the UniProt Knowledgebase. We then identified potential common targets by intersecting the screened TNBC-related disease targets (DEGs) with the candidate BBR drug targets. This multi-omics integration strategy aims to identify core therapeutic nodes that are both transcriptionally dysregulated in the TNBC disease state (indicating pathological relevance) and possess structural binding pockets for pharmacological intervention (indicating druggability) (Shi et al., 2024; Ma et al., 2025). The overlapping targets were considered candidate therapeutic targets through which BBR may regulate TNBC. Subsequently, these targets were imported into the STRING database (species restricted to *Homo sapiens*) to construct a protein–protein interaction (PPI) network, with the minimum required interaction confidence score set at 0.4 (Szklarczyk et al., 2017). The constructed PPI network was then imported into Cytoscape software (v3.10.0) for visualization, and the CytoNCA plugin was used to identify key nodes based on multiple network topological parameters, including local average connectivity (LAC), degree centrality (DC), betweenness centrality (BC), closeness centrality (CC), eigenvector centrality (EC), and network centrality (NC) (Xiang et al., 2019). The median value of each parameter was used as the screening threshold, and core targets within the PPI network were ultimately identified through multiple rounds of filtering.

### 2.7 GO and KEGG enrichment analysis

Functional annotation analysis of the screened candidate therapeutic targets was conducted using the clusterProfiler package in R (Yu et al., 2012). Gene Ontology (GO) analysis encompassed three categories: biological process (BP), cellular component (CC), and molecular function (MF). In addition, Kyoto Encyclopedia of Genes and Genomes (KEGG) pathway enrichment analysis was performed to identify significantly enriched signaling pathways. For all enrichment analyses, a significance threshold of P < 0.05 was applied. The significantly enriched pathways were visualized, and the distribution of candidate targets within the relevant signaling pathways was annotated using KEGG Mapper.

### 2.8 Immune infiltration analysis

Based on the GSE31488, GSE45827, and GSE65194 datasets, the CIBERSORT algorithm was applied to estimate the relative proportions of 22 immune cell subsets in each sample (Newman et al., 2015). To ensure the reliability of the deconvolution results, a p-value was calculated for each sample using 1,000 permutations. Only samples with a CIBERSORT output P < 0.05 were considered to have a significant and credible immune cell infiltration profile and were included in the subsequent comparative and correlation analyses. The immune infiltration results were visualized using heatmaps and boxplots to compare differences between normal breast tissues and TNBC tissues. The corrplot package in R was used to analyze and display the correlations among different immune cell subsets.Furthermore, Pearson correlation analysis was performed to assess the relationship between the expression levels of core therapeutic targets and the degree of immune cell infiltration, and the results were visualized using the ggplot2 package.

### 2.9 scRNA-seq analysis

Single-cell RNA sequencing data were obtained from the GEO database (GSE161529), comprising six TNBC samples. The Seurat package in R was used to construct the object and perform subsequent analyses. The quality control criteria were as follows: each gene expressed in at least 3 cells; each cell containing 200–5,000 detected genes; and the proportions of mitochondrial and ribosomal genes not exceeding 20%. After normalization using the LogNormalize method, 1,500 highly variable genes were identified and processed with the ScaleData function. Principal component analysis (PCA) was performed based on these highly variable genes, and the Harmony method was applied to correct batch effects among different samples (Satija et al., 2015). Subsequently, a nearest-neighbor graph was constructed, clustering analysis was conducted using the Louvain algorithm, and dimensionality reduction was visualized using t-SNE. Cell type annotation was performed using SingleR with reference to multiple publicly available human cell databases. The irGSEA method was used to calculate the activity distribution of candidate therapeutic targets across different cell subpopulations, and the results were presented using heatmaps, bubble plots, and feature plots.

### 2.10 Molecular docking and molecular dynamics simulation

The two-dimensional chemical structure of BBR was obtained from the PubChem database and geometrically optimized prior to molecular docking. The three-dimensional structures of candidate target proteins were retrieved from the UniProt and Protein Data Bank (PDB) databases and subjected to standardized preprocessing before docking, including removal of crystallographic water molecules and original ligands, addition of hydrogen atoms, and structural correction. A grid box was constructed centered on the predicted active site, molecular docking was performed using AutoDock Vina, and the binding energies of the ligand–protein complexes were recorded. The conformation with the lowest binding energy was selected for subsequent analysis and visualized using PyMOL (Forli et al., 2016). Subsequently, molecular dynamics simulations were conducted using GROMACS 2022. The protein was described using the AMBER14SB force field, the system was placed in a dodecahedral box and solvated with the TIP3P water model, followed by energy minimization and equilibration under NVT and NPT ensembles (Mark and Nilsson, 2001). Finally, a 100 ns molecular dynamics simulation was performed, and the structural stability and conformational changes of the system were analyzed.

### 2.11 Cell culture

The TNBC cell lines HCC1937 and MDA-MB-231 were cultured in RPMI-1640 and high-glucose DMEM media, respectively. The media were supplemented with 10% fetal bovine serum and 1% penicillin–streptomycin, and cells were maintained in a humidified incubator at 37 °C with 5% CO_2_. The culture medium was replaced every 2–3 days according to cell growth conditions. When cell confluence reached approximately 80%, cells were passaged and used for subsequent experiments.

### 2.12 Cell proliferation assays

Cell proliferation was evaluated using the CCK-8 assay. HCC1937 and MDA-MB-231 cells in the logarithmic growth phase were routinely digested and seeded into 96-well plates at a density of 5 × 10^3^ cells per well. After 24 h of incubation, cells were treated with different concentrations of BBR (0, 20, 40, 80, 120, and 160 μmol/L) for 24,48, and 72 h, with a blank control group included. Following treatment, 10 μL of CCK-8 reagent was added to each well and incubated at 37 °C for 1 h in the dark. The optical density (OD) was measured at 450 nm using a microplate reader, and the cell proliferation rate and half-maximal inhibitory concentration (IC_50_) were calculated accordingly.

The EdU incorporation assay was performed according to the manufacturer’s instructions to assess cell proliferation. HCC1937 cells were seeded into 24-well plates at a density of 5 × 10^3^ cells per well. After 24 h of incubation, cells were treated with BBR for 48 h, and 10 μM EdU was added simultaneously for 2 h of incubation. After treatment, cells were fixed with 4% paraformaldehyde for 15 min, and staining was performed according to the kit protocol. The proportion of EdU-positive cells was calculated using ImageJ software to evaluate the level of cell proliferation.

In the colony formation assay, HCC1937 cells in the logarithmic growth phase were seeded into 6-well plates at a density of 1 × 10^3^ cells per well. After 24 h, cells were treated with different concentrations of BBR and cultured for 14 days in RPMI-1640 medium containing 10% FBS to allow colony formation. At the end of incubation, cells were washed with cold PBS, fixed with 4% paraformaldehyde for 15 min, and stained with 1% crystal violet for 15 min. Finally, the number of colonies in each well was quantified using ImageJ software.

### 2.13 Cell apoptosis assay

HCC1937 cells were seeded into 6-well plates at a density of 5 × 10^5^ cells per well and treated with different concentrations of BBR for 48 h. To investigate the role of ROS, a subset of cells was pre-treated with 5 mM N-acetylcysteine (NAC) for 2 h prior to BBR exposure. After treatment, cells were collected, washed with PBS, and resuspended in 500 μL of 1× Binding Buffer. Subsequently, 5 μL Annexin V-APC and 10 μL 7-AAD were added (with single-stained compensation controls prepared separately), gently mixed, and incubated at room temperature in the dark for 5 min.The apoptosis rate was then analyzed using flow cytometry.

### 2.14 Detection of ROS levels

Intracellular reactive oxygen species (ROS) levels were measured using the fluorescent probe 2′,7′-dichlorodihydrofluorescein diacetate (DCFH-DA). HCC1937 cells were seeded into 6-well plates, treated with different concentrations of BBR and then washed twice with serum-free medium. The DCFH-DA probe was diluted in serum-free medium to a final concentration of 10 μM, added to the cells, and incubated at 37 °C for 30 min in the dark. After incubation, the excess probe was discarded, and the cells were washed with PBS. Fluorescence signals were observed under a fluorescence microscope, or fluorescence intensity was measured by flow cytometry to assess changes in intracellular ROS levels.

### 2.15 Effect of BBR on TNBC stemness

To evaluate the effect of BBR on the stemness of HCC1937 cells, sphere formation assays and CD133 flow cytometry analysis were performed. For the tumor sphere formation assay, HCC1937 cells in the logarithmic growth phase were seeded at a density of 4,000 cells per well in ultra-low attachment 24-well plates. Cells were cultured in 1 mL of MammoCult™ specialized medium (pre-supplemented with 0.2% heparin and 0.5% hydrocortisone) containing different concentrations of BBR (0, 20, 40, and 80 μM). Sphere formation was observed daily, and once the morphology stabilized, ImageJ software was used to count and measure spheres with a diameter ≥50 μm. The sphere volume was calculated using the formula V = 4/3πr^3^. Meanwhile, cells cultured and treated in 6-well plates were collected and incubated with PE-conjugated CD133 antibody at 4 °C in the dark for 30 min. After washing with PBS, the proportion of CD133-positive cells was analyzed by flow cytometry. A blank control group was used to eliminate nonspecific signals, thereby evaluating changes in the TNBC stem-like subpopulation.

### 2.16 Western blot analysis

Total protein was extracted from BBR-treated HCC1937 cells (cultured in 6-well plates) using RIPA lysis buffer containing protease and phosphatase inhibitors. For the ROS rescue experiments, cells were pre-treated with 5 mM NAC for 2 h before the addition of BBR. After centrifugation, the supernatant was collected and protein concentration was determined. Equal amounts of protein were separated by 10% SDS-PAGE and subsequently transferred onto PVDF membranes. Membranes were blocked with 5% non-fat milk at room temperature for 1 h and then incubated overnight at 4 °C with primary antibodies against GAPDH, BAX, BCL-2, cleaved caspase-3, AKT, p-AKT, PI3K, p-PI3K, SRC, p-SRC, STAT3, and p-STAT3. After washing, membranes were incubated with HRP-conjugated secondary antibodies for 1 h, and protein bands were visualized using an enhanced chemiluminescence (ECL) detection system.Band intensities were quantified using ImageJ software. Protein expression levels were normalized to GAPDH, and phosphorylation levels were expressed as the ratios of p-AKT/AKT and p-PI3K/PI3K.

### 2.17 Statistical analysis

All data were analyzed using GraphPad Prism 10.6.0 and R software. Results are presented as mean ± standard deviation (mean ± SD). Comparisons between two groups were performed using Student’s t-test, while comparisons among multiple groups were conducted using one-way analysis of variance (one-way ANOVA). Followed by Dunnett’s *post hoc* test to evaluate the statistical significance between each treatment group and the control group. A P value < 0.05 was considered statistically significant.

## 3 Results

### 3.1 Drug targets of BBR

**FIGURE 1:**
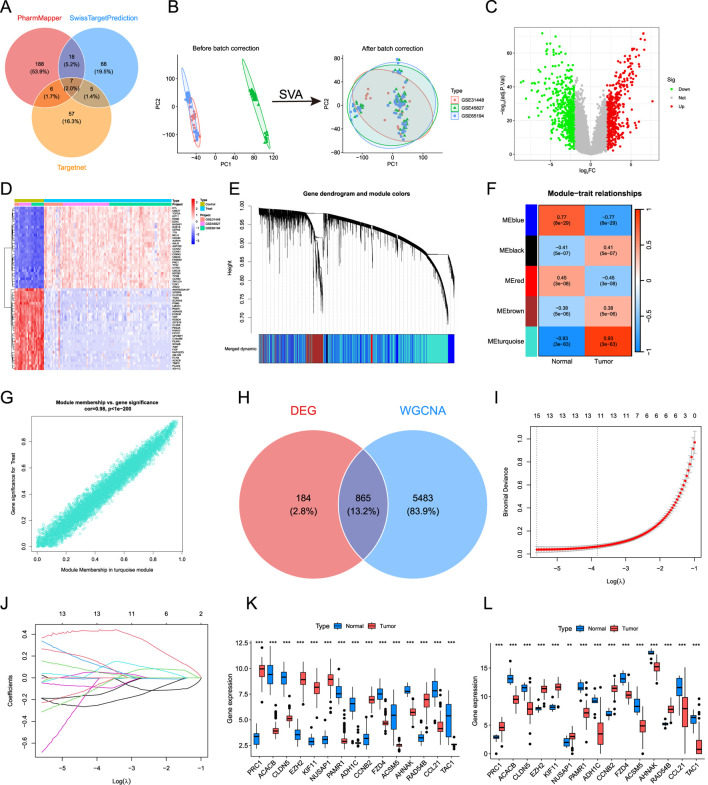
Target screening for BBR and TNBC **(A)** Venn diagram of BBR targets predicted from three databases **(B)** Principal component analysis (PCA) plots before and after batch correction **(C)** Volcano plot of DEGs **(D)** Heatmap of the expression of the top 60 DEGs **(E)** Dendrogram of gene clustering **(F)** Heatmap of module-trait relationships **(G)** Correlation between the turquoise module eigengene and TNBC **(H)** Venn diagram showing the intersection of DEGs and genes in the turquoise module **(I)** LASSO regression model **(J)** Adjustment of feature selection in the minimum absolute shrinkage **(K)** Comparison of candidate gene expression in the GSE datasets **(L)** Validation of candidate gene expression in the TCGA database.

A total of 98 and 75 potential BBR-related targets were predicted using the SwissTargetPrediction and TargetNet platforms, respectively. In addition, 219 potential drug-related targets were obtained from the PharmMapper database. After integrative analysis and removal of duplicate targets, a total of 349 drug-related targets were ultimately identified (Figure 1A; Supplementary Material S2).

### 3.2 Identification of TNBC DEGs

After integrating the GEO datasets and correcting for batch effects, principal component analysis (PCA) demonstrated a clear separation between tumor tissues and normal breast tissues in terms of gene expression profiles (Figure 1B). A total of 1,049 differentially expressed genes (DEGs) were identified, including 500 upregulated genes and 549 downregulated genes (Supplementary Material S3). The volcano plot and hierarchical clustering heatmap further illustrated the expression patterns of these genes across different samples (Figures 1C,D).

### 3.3 WGCNA and identification of key genes

A weighted gene co-expression network was constructed based on the batch-corrected transcriptomic data. By evaluating different soft-thresholding powers, β = 3 was selected as the optimal parameter to ensure that the network satisfied the scale-free topology criterion (*R* ^*2*^ ≥ 0.8) (Supplementary Figure S1). A topological overlap matrix (TOM) was then constructed, and five co-expression modules were identified using the dynamic tree cut algorithm (Figure 1E). Module–trait relationship analysis revealed that the turquoise module was highly positively correlated with the TNBC phenotype (cor = 0.93, *P* = 3e-63) (Figure 1F). This module contained 6,348 genes strongly associated with triple-negative breast cancer (cor = 0.98, *P* < 1e-200) (Figure 1G). Intersection analysis between module genes and DEGs, followed by removal of duplicates, yielded 865 candidate genes (Figure 1H). Subsequently, LASSO regression analysis identified 15 feature genes (Figures 1I,J). The differential expression of these genes between control and TNBC groups was validated in the TCGA dataset (Figures 1K,L), and their discriminative performance was evaluated using ROC curve analysis (Supplementary Figures S2A,B). Finally, these 15 feature genes were integrated with 2,830 previously reported TNBC-related genes collected from the GeneCards, OMIM, and TTD databases, resulting in a total of 2,835 TNBC-associated genes (Supplementary Material Figure S4).

### 3.4 PPI target analysis of BBR against TNBC

**FIGURE 2:**
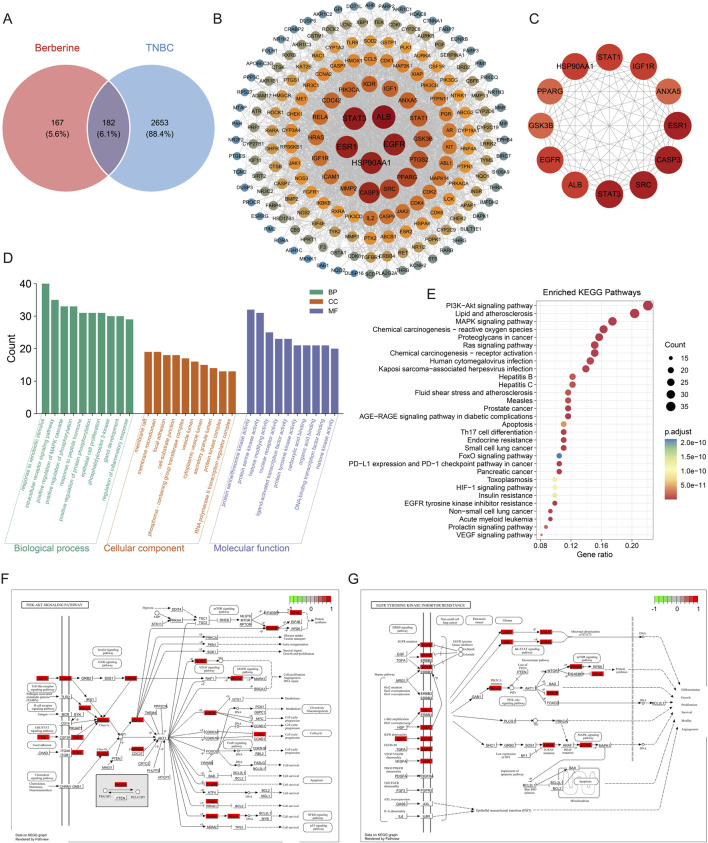
PPI network and enrichment analysis of BBR anti-TNBC targets. **(A)** Venn diagram of BBR anti-TNBC targets. **(B)** PPI network of BBR anti-TNBC targets. **(C)** Core genes identified by CytoNCA analysis. **(D)** GO biological process functional annotation. **(E)** Enriched KEGG pathways. **(F)** Core PI3K–AKT signaling pathway based on KEGG (therapeutic targets are marked in red). **(G)** Pathway mapping of the core genes.

Intersection analysis between 2,835 TNBC-related genes and 349 candidate BBR drug targets identified 182 overlapping genes (Figure 2A). These genes were subsequently included in protein–protein interaction (PPI) network analysis. The PPI network constructed based on the STRING database consisted of 182 nodes and 2,496 interaction edges, with an average node degree of 27.4 and an average local clustering coefficient of 0.531. The PPI enrichment *P* < 1.0e-16. The PPI network was visualized using Cytoscape, and screening analysis was performed using the CytoNCA plugin based on multiple topological parameters.After multiple rounds of topological parameter filtering, 12 highly central key genes were ultimately identified, including STAT3, CASP3, SRC, EGFR, and ESR1 (Figures 2B,C).

### 3.5 GO and KEGG enrichment analysis

GO functional annotation and KEGG pathway enrichment analyses were performed on the screened candidate therapeutic targets. Gene Ontology biological process (BP) analysis indicated that these genes were mainly involved in cell proliferation, apoptosis, angiogenesis, and cell migration. At the molecular function (MF) level, the associated genes were mainly enriched in protein binding, transcription factor binding, and kinase activity (Figure 2D). KEGG pathway enrichment analysis revealed that the candidate targets were predominantly enriched in the PI3K–Akt, MAPK, and TNF signaling pathways, and were closely associated with oxidative stress, infection, and metabolism-related pathways (Figure 2E). Among these, the PI3K–Akt signaling pathway showed the highest degree of enrichment. Subsequently, KEGG Mapper was used to map the PI3K–Akt and other related pathways, and the distribution of candidate targets within the corresponding pathways was annotated (Figures 2F,G).

### 3.6 Immune infiltration analysis

**FIGURE 3:**
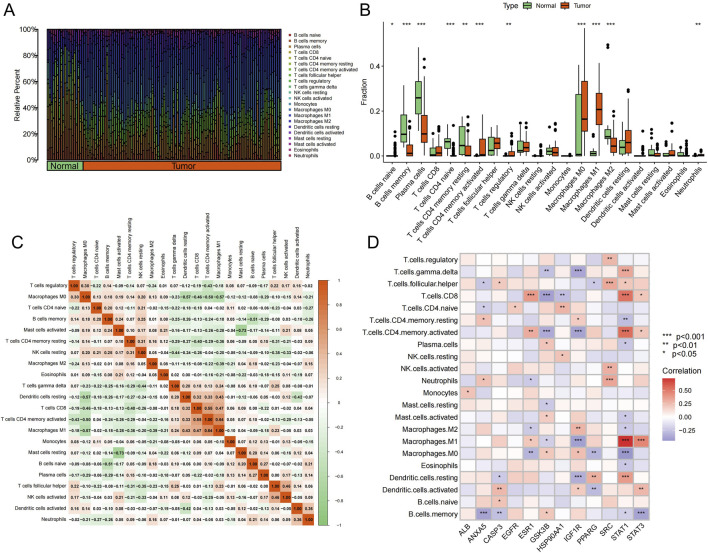
Analysis of immune infiltration characteristics in TNBC. **(A)** Heatmap and **(B)** boxplot showing the distribution of the infiltration levels of 22 immune cell types and the differences between groups in the GSE31488, GSE45827, and GSE65194 datasets, respectively. **(C)** Correlation analysis among the 22 immune cell types. **(D)** Correlation analysis between the 12 key targets and the 22 immune cell types.

The relative infiltration levels of 22 immune cell subsets were evaluated in the GSE31488, GSE45827, and GSE65194 datasets. The results showed significant differences in the infiltration proportions of multiple immune cell subsets between TNBC tissues and normal breast tissues (*P* < 0.05) (Figures 3A,B). The immune cell infiltration heatmap further illustrated the distribution characteristics of each immune cell subset across different samples (Figure 3C). Further analysis was conducted to examine the correlations between the hub genes and immune cell infiltration. The results indicated that SRC was positively correlated with neutrophils and follicular helper T cells; STAT3 was negatively correlated with memory B cells, while being positively correlated with M1 macrophages; GSK3B, IGF1R, and STAT1 were significantly correlated with multiple immune cell subsets, whereas ALB and EGFR showed weaker correlations with immune cell infiltration (Figure 3D).

### 3.7 Analysis of cellular distribution characteristics of hub genes by scRNA-seq

**FIGURE 4:**
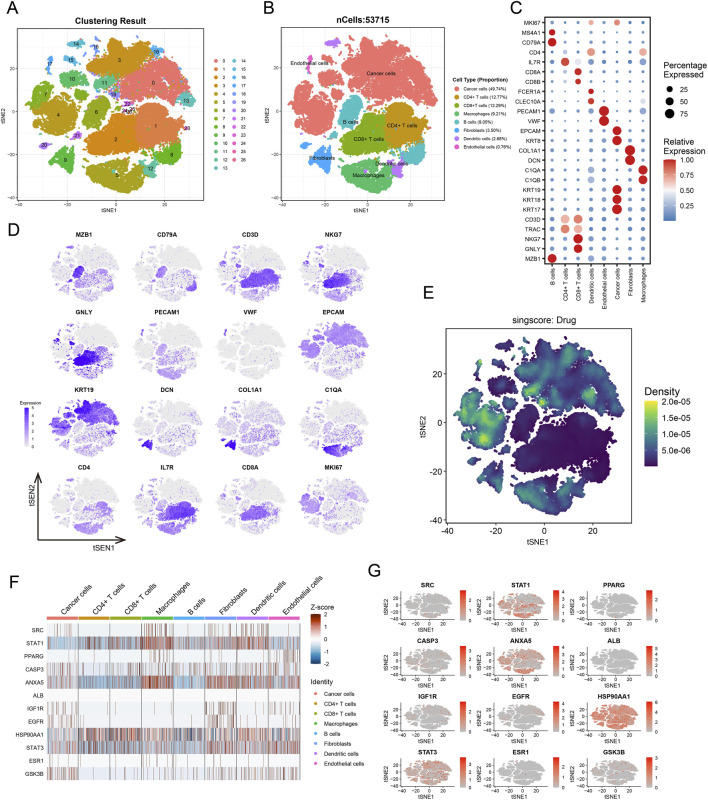
Elucidation of BBR-targeted cell types by scRNA-seq. **(A)** t-SNE plot showing 26 cell clusters. **(B)** Cell type annotation based on marker genes. **(C)** Dot plot displaying the expression of canonical marker genes. **(D)** t-SNE distribution of key marker genes. **(E)** Activity distribution of BBR targets across cell types. **(F)** Heatmap showing the expression of 12 core genes in eight cell subpopulations. **(G)** t-SNE expression distribution of the 12 key genes.

Following quality control and downstream analysis of single-cell RNA sequencing data from triple-negative breast cancer, a total of 53,715 high-quality cells were obtained. Clustering analysis was performed using Seurat (resolution = 0.6), which identified 26 distinct cell clusters (Figure 4A). Based on canonical marker genes, these cells were annotated as eight major cell types, including epithelial cells/tumor cells, CD4^+^ T cells, CD8^+^ T cells, macrophages, B cells, fibroblasts, dendritic cells, and endothelial cells (Figure 4B). The marker genes for each cell type exhibited specific expression patterns, further validating the accuracy of the cell annotations (Figures 4C,D). The irGSEA method was employed to evaluate the activity distribution of candidate therapeutic target genes across different cell types. The results indicated that the activity of these genes was primarily concentrated in tumor cell and macrophage populations (Figure 4E). The expression patterns of the candidate hub genes across different cell clusters were visualized using heatmaps and t-SNE plots (Figures 4F,G).

### 3.8 Binding affinity analysis

**FIGURE 5:**
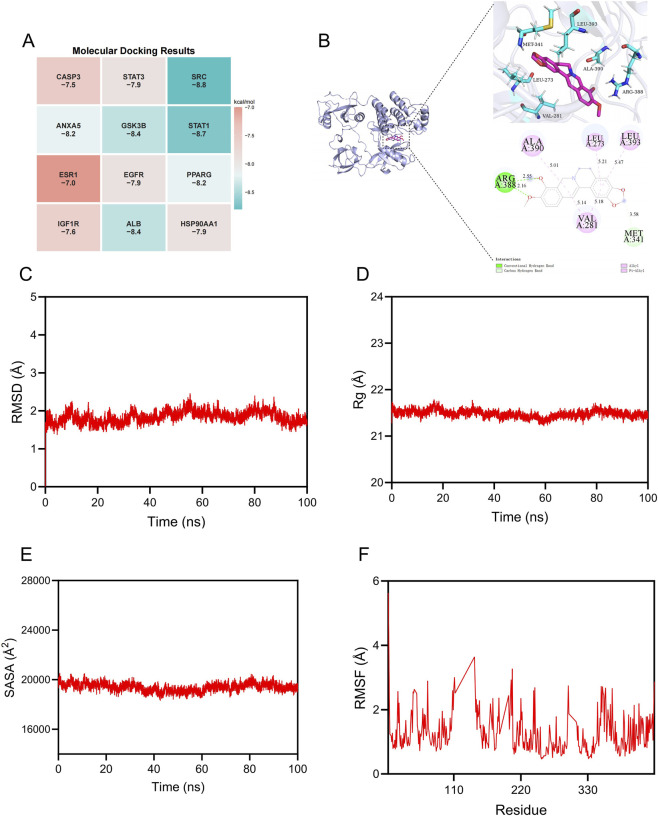
Binding affinity analysis. **(A)** Heatmap of binding energies between the 12 hub genes and BBR. **(B)** Molecular docking pattern of BBR with the SRC protein. **(C)** RMSD fluctuation of the protein-ligand complex. **(D)** Rg fluctuation of the protein-ligand complex. **(E)** SASA fluctuation of the protein-ligand complex. **(F)** RMSF analysis of the protein-ligand complex.

Molecular docking results showed that the binding energies of BBR with the 12 candidate targets were all lower than −5 kcal/mol (Figure 5A). Among these, BBR exhibited the lowest binding energy with SRC (−8.8 kcal/mol). To evaluate the binding potency of BBR, we performed parallel docking with Dasatinib, a well-established potent SRC kinase inhibitor, as a positive control. The docking results revealed that BBR possesses a strong binding capacity nearly comparable to that of Dasatinib (−9.1 kcal/mol), as shown in Supplementary Figure S4. The molecular docking conformation indicated that BBR could embed into the binding pocket of the SRC protein and form a stable binding conformation (Figure 5B). Detailed analysis of the docking model revealed that BBR binds precisely within the highly conserved ATP-binding pocket of the SRC kinase domain, which is the same region typically occupied by the original co-crystallized ligands (such as Dasatinib) and ATP. Specifically, BBR forms a stable conventional hydrogen bond with ARG388. Furthermore, the molecular scaffold of BBR is stabilized by extensive hydrophobic interactions with key surrounding residues, including LEU273, VAL281, MET341, ALA390, and LEU393. Notably, 2D interaction analysis confirmed that BBR and Dasatinib share several critical binding residues, including LEU273, ARG388, and ALA390, further indicating that BBR effectively targets the functional active site of the SRC kinase. The occupancy of this pocket by BBR likely impedes ATP binding, thereby inhibiting SRC catalytic activity.

Molecular dynamics simulation results demonstrated that the complex reached equilibrium after 10 ns and subsequently fluctuated stably within a 1.8 Å range (Figure 5C), indicating high stability. Analysis of the radius of gyration (Rg) and solvent-accessible surface area (SASA) indicated that the overall structure of the complex was stable, and ligand binding had a minor impact on the protein conformation (Figures 5D,E). Root mean square fluctuation (RMSF) analysis further revealed that the complex exhibited low overall flexibility and maintained high stability (Figure 5F). Comprehensive analysis suggested that BBR possesses strong binding stability with SRC, and the complex maintained stable structural fluctuations throughout the simulation process.

### 3.9 BBR inhibits cell proliferation and induces apoptosis

**FIGURE 6:**
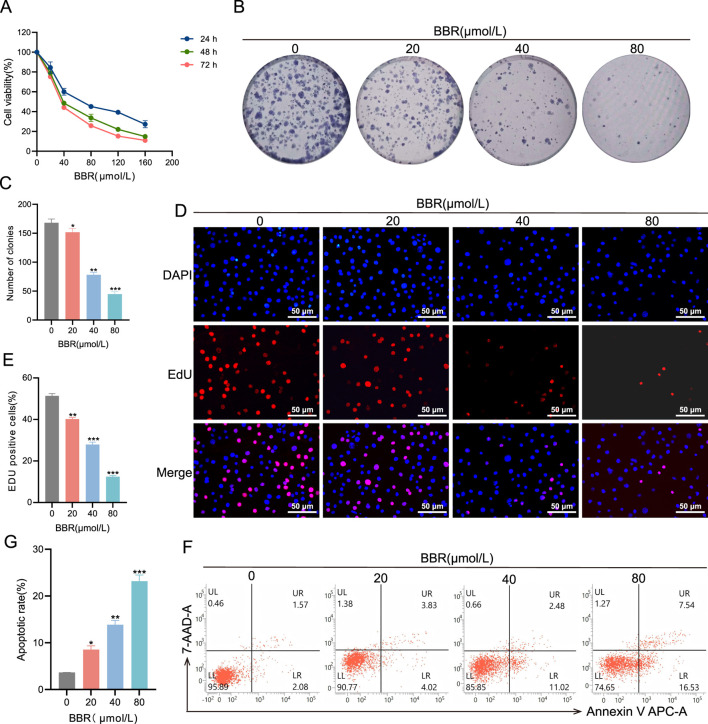
BBR inhibits proliferation and induces apoptosis in HCC1937 cells. **(A)** Cell viability detected by CCK-8 assay. **(B)** Colony formation assay. **(C)** Quantification of colony formation. **(D)** Cell proliferation detected by EdU assay. **(E)** Percentage of EdU-positive cells. **(F)** Apoptosis detected by flow cytometry. **(G)** Quantification of apoptosis rate. All experiments were repeated three times. Scale bars = 50 μm *p < 0.05, **p < 0.01, ***p < 0.001.

To evaluate the effect of BBR on the proliferation of triple-negative breast cancer (TNBC) cells, MDA-MB-231 and HCC1937 cells were treated with varying concentrations of Berberine for 24, 48, and 72 h. CCK-8 assay results showed that the viability of both cell types decreased significantly following BBR treatment in a time-dependent and concentration-dependent manner. IC_50_ analysis indicated that the IC_50_ values for BBR treatment on HCC1937 cells at 24, 48, and 72 h were 60.50 μM, 41.89 μM, and 37.92 μM, respectively; at the same time points, the IC_50_ values for MDA-MB-231 cells were similar to those for HCC1937 cells (Figure 6A; Supplementary Figure S3). Colony formation assay results revealed that the number of colonies formed by HCC1937 cells was significantly reduced after BBR treatment (Figures 6B,C). EdU proliferation assay results demonstrated that the proportion of EdU-positive cells in the BBR-treated group was significantly lower than that in the control group (Figures 6D,E). Furthermore, apoptosis was analyzed using Annexin V-APC/7-AAD double staining flow cytometry. The results showed that after 48 h of BBR treatment, the proportion of apoptotic cells increased significantly with increasing drug concentration (Figures 6F,G).

### 3.10 BBR induces ROS accumulation accompanied by inhibition of the PI3K/AKT pathway and altered expression of apoptosis-related proteins

**FIGURE 7:**
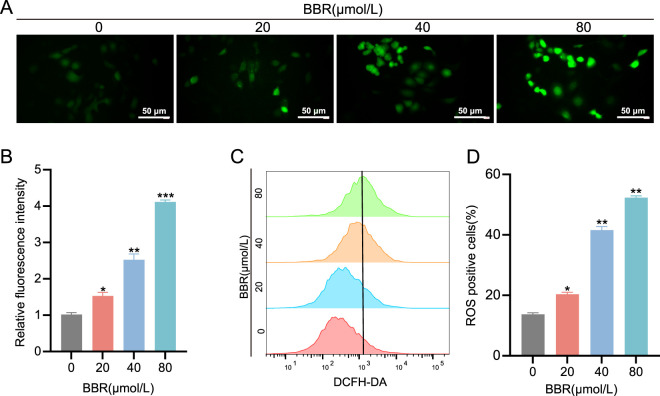
Effect of BBR on ROS production. **(A)** Detection of ROS levels by fluorescence microscopy. **(B)** Quantification of ROS fluorescence intensity. **(C)** Detection of ROS by flow cytometry. **(D)** Quantification of the proportion of ROS-positive cells. All experiments were repeated three times. Scale bar = 50 μm *p < 0.05, **p < 0.01, ***p < 0.001.

ROS are important signaling molecules that can mediate oxidative stress and cause cellular damage when their regulation is imbalanced. KEGG enrichment analysis suggested that ROS accumulation might be associated with the anti-tumor effects of BBR against TNBC. Therefore, the DCFH-DA fluorescent probe was used to detect changes in ROS levels in HCC1937 cells following treatment with different concentrations of BBR. The results showed that intracellular ROS levels in HCC1937 cells were significantly increased after BBR treatment, exhibiting a concentration-dependent rise, which further exacerbated cellular oxidative stress (Figures 7A–D).

**FIGURE 8:**
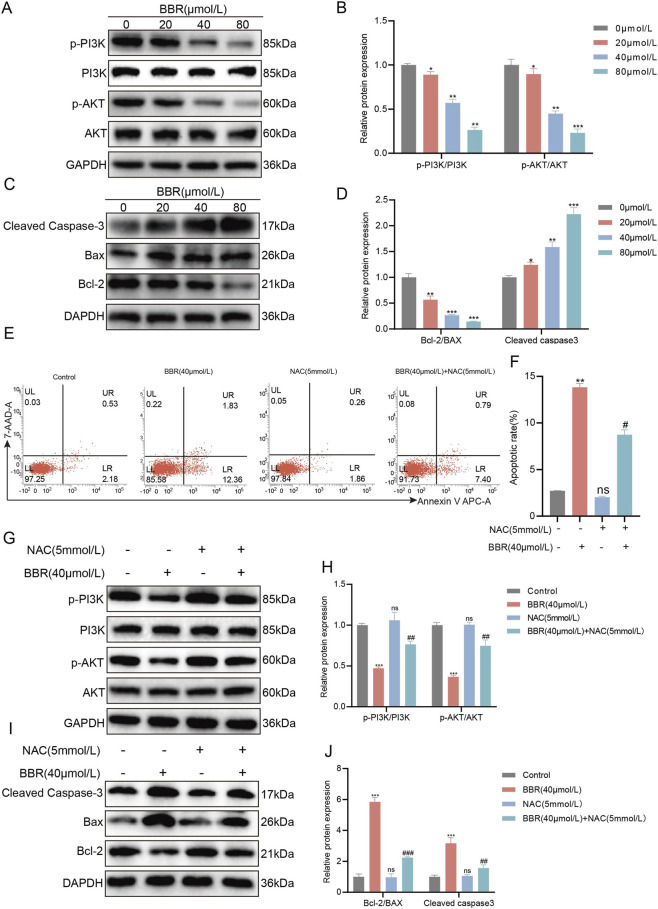
The ROS/PI3K/AKT axis mediates BBR-induced apoptosis **(A,B)** Western blot analysis and quantification of p-PI3K, PI3K, p-AKT, and AKT expression **(C,D)** Western blot analysis and quantification of BAX, BCL-2, and Cleaved Caspase-3 expression **(E,F)** Detection and quantification of apoptosis by flow cytometry **(G,H)** Expression and quantification of p-PI3K and p-AKT after NAC pre-treatment **(I,J)** Expression and quantification of BAX, BCL-2, and Cleaved Caspase-3 after NAC pre-treatment. All experiments were repeated three times. Compared with the control group: ns indicates no significant difference, *p < 0.05, **p < 0.01, ***p < 0.001; Compared with the BBR (40 μmol/L) group: #*p* < 0.05, ##*p* < 0.01, ###*p* < 0.001.

Given that KEGG enrichment analysis suggested the potential involvement of the PI3K/AKT signaling pathway in the biological effects of BBR, the expression changes of proteins in this pathway and its downstream apoptosis-related proteins were further examined. Western blot results showed that after BBR treatment, the protein levels of p-PI3K and p-AKT gradually decreased with increasing concentration, while the expression of total PI3K and total AKT remained largely stable (Figures 8A,B), suggesting that BBR primarily inhibits the phosphorylation level of this pathway. Concurrently, the expression of pro-apoptotic proteins BAX and Cleaved Caspase-3 was upregulated, while the expression of the anti-apoptotic protein BCL-2 was downregulated, and these changes exhibited a dose-dependent trend (Figures 8C,D). To further clarify the involvement of ROS in the aforementioned molecular events, cells were pre-treated with the ROS scavenger N-acetylcysteine (NAC) prior to BBR treatment. Functional assay results showed that NAC significantly attenuated the apoptosis rate induced by BBR (Figures 8E,F). These results indicate that BBR treatment is accompanied by increased ROS levels, decreased phosphorylation levels of the PI3K/AKT pathway (Figures 8G,H), and remodeling of apoptosis-related protein expression; whereas ROS scavenging partially reversed these molecular changes and the apoptotic phenotype (Figures 8I,J).

### 3.11 BBR targets the SRC/STAT3 pathway to inhibit stemness in TNBC cells

**FIGURE 9:**
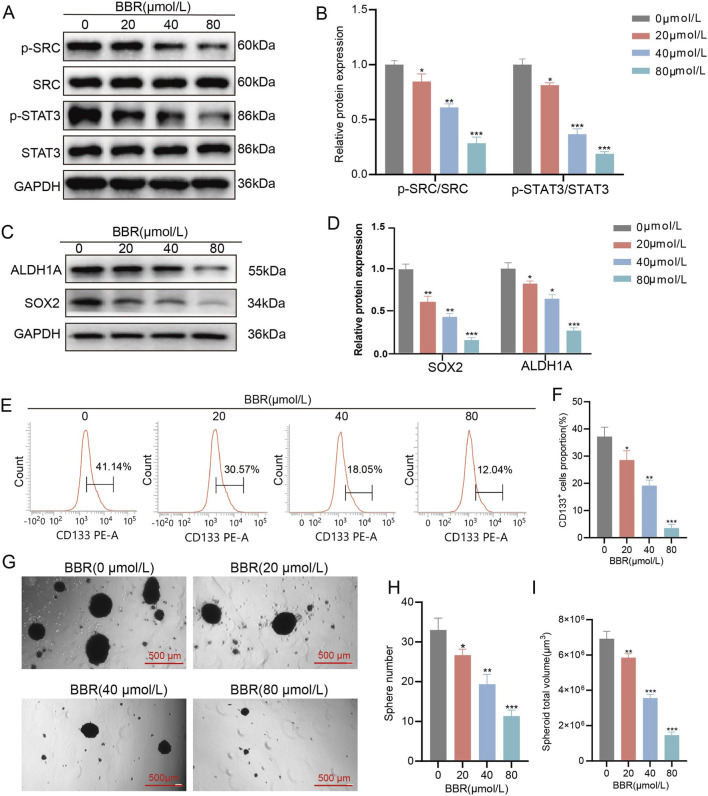
BBR targets the SRC/STAT3 pathway to inhibit stemness in TNBC cells **(A,B)** Western blot analysis and quantification of p-SRC, SRC, p-STAT3, and STAT3 expression **(C,D)** Western blot analysis and quantification of canonical stemness markers ALDH1A and SOX2 **(E)** Flow cytometry histogram showing the proportion of CD133^+^ cells **(F**) Quantification of the proportion of CD133^+^ cells **(G)** Representative images of tumorsphere formation **(H)** Quantification of the number of tumorspheres **(I)** Quantification of tumorsphere size. All experiments were repeated three times. Scale bar = 500 μm *p < 0.05, **p < 0.01, ***p < 0.001.

Based on the predictions from molecular docking and molecular dynamics simulations, the interaction between BBR and SRC was further validated experimentally. Western blot results showed that BBR significantly inhibited the phosphorylation of SRC at its activation site (Tyr416) and downregulated the phosphorylation level of the downstream signaling molecule STAT3 (Figures 9A,B). To comprehensively assess the effect of BBR on cancer stem cell properties, we evaluated multiple classical stemness markers alongside a functional tumorsphere formation assay. Western blot analysis revealed that BBR significantly downregulated the protein expression of canonical stemness markers, including ALDH1A and the core pluripotency transcription factor SOX2, in a dose-dependent manner (Figures 9C,D). Consistently, flow cytometry analysis demonstrated that the proportion of the CD133^+^ cell subpopulation in TNBC cells prominently decreased with increasing drug concentration (Figures 9E,F). Furthermore, the functional tumorsphere formation assay showed that both the number and size of tumorspheres were significantly reduced following BBR treatment (Figures 9G–I). These findings indicate that BBR attenuates the stemness maintenance capacity of TNBC cells by inhibiting SRC activation and blocking the SRC/STAT3 signaling pathway.

## 4 Discussion

Triple-negative breast cancer is the most aggressive subtype of breast cancer and is associated with a poor prognosis. Current treatment options are limited to surgery, adjuvant chemotherapy, and radiotherapy (Li et al., 2022; Wang et al., 2024). Currently, chemotherapy based on taxanes and anthracyclines remains the standard treatment regimen for TNBC; however, its efficacy is often limited by significant systemic toxicity, acquired drug resistance, and the risk of tumor recurrence, which severely impacts patients’ long-term survival benefits (Lee, 2023; Serrano et al., 2024; Sharma et al., 2024). Therefore, developing novel, safe therapeutic strategies that can simultaneously modulate multiple oncogenic signaling pathways is clinically significant. In this context, multi-targeted drugs derived from natural products have garnered widespread attention in recent years due to their ability to synergistically intervene in complex tumor signaling networks (Yang et al., 2021; Zhong et al., 2022).

Berberine (BBR), an isoquinoline alkaloid derived from various traditional Chinese herbs, has been demonstrated to possess multiple pharmacological activities, including anti-inflammatory, antioxidant, metabolic regulatory and anti-tumor activities (Xiong et al., 2022; Mohammadian et al., 2025; Shi et al., 2025). Previous studies have indicated that BBR can inhibit the occurrence and progression of various tumor types by regulating multiple signaling pathways, such as PI3K/AKT, JAK/STAT, and oxidative stress pathways (Khezri et al., 2024; Long et al., 2025). However, its systematic pharmacological mechanism of action in TNBC, particularly its potential regulatory effects on tumor cell heterogeneity and the tumor microenvironment, remains to be further elucidated.

In this study, by integrating the prediction results from platforms such as PharmMapper and SwissTargetPrediction with TNBC transcriptomic data from GEO and TCGA, we identified 182 overlapping targets between BBR and TNBC. Functional enrichment analysis revealed that these key targets were primarily enriched in classical tumor-related signaling pathways, such as PI3K/AKT and JAK/STAT, which play critical roles in the proliferation, survival, and stemness maintenance of TNBC cells (Glaviano et al., 2023; Long et al., 2024; Zhang et al., 2024). Further protein-protein interaction network analysis and topological screening (based on parameters such as degree centrality and betweenness centrality) identified 12 key hub genes, including SRC, STAT3, CASP3, and EGFR. Notably, these genes have been widely demonstrated to play central roles in regulating tumor cell proliferation, survival, and apoptosis, and are closely associated with tumor progression and therapeutic resistance (Poh and Ernst, 2023; Fasouli and Katsantoni, 2024; Malekinejad et al., 2025). Molecular docking results showed that the binding energies of BBR with the key targets were all lower than −5.0 kcal/mol. Among these, BBR exhibited the lowest binding energy with SRC (−8.8 kcal/mol). Our molecular docking results demonstrated that BBR securely embeds into the highly conserved ATP-binding pocket of the SRC kinase domain, a critical region for its phosphorylation and downstream signaling. By competing for the same binding site as ATP and forming stable interactions with key hinge and hydrophobic cleft residues (including a conventional hydrogen bond with ARG388 and extensive hydrophobic interactions with LEU273, VAL281, MET341, ALA390, and LEU393), BBR effectively acts as an ATP-competitive inhibitor. Notably, BBR exhibited a strong binding affinity comparable to that of the classical SRC inhibitor, dasatinib, further underscoring its potent targeted inhibitory capacity. Furthermore, the 100 ns molecular dynamics (MD) simulation trajectory confirmed the continuous stability of the BBR-SRC complex, providing a robust computational biology basis for the direct action of BBR on SRC. Ultimately, this structural evidence perfectly rationalizes our subsequent experimental observations that BBR treatment drastically downregulates p-SRC levels in TNBC cells.

Furthermore, through immune infiltration analysis and single-cell transcriptome sequencing, the mechanism of action of BBR was extended to the level of the tumor microenvironment. CIBERSORT analysis based on multiple GEO cohorts revealed significant differences in immune cell infiltration patterns between TNBC tissues and normal tissues. Correlation analysis further revealed that SRC was positively correlated with neutrophils and follicular helper T cells, while STAT3 was negatively correlated with memory B cells and positively correlated with M1 macrophages. This finding suggests that the signaling pathways targeted by BBR not only function in tumor cells but may also participate in shaping the immunosuppressive microenvironment. The SRC/STAT3 pathway has been reported to regulate the expression of PD-L1 on tumor cells and the polarization of macrophages (Rébé and Ghiringhelli, 2019; Shi et al., 2021; Saranyutanon et al., 2022). Therefore, the inhibition of SRC/STAT3 by BBR could potentially influence the cancer-immunity cycle, providing a theoretical rationale for its combined application with immune checkpoint inhibitors.

Single-cell RNA sequencing analysis provided a high-resolution perspective on the cell-specific distribution of BBR targets. Through in-depth analysis of single-cell data from TNBC tissues, we successfully identified major cell populations, including tumor cells, T cells, B cells, macrophages, and fibroblasts. IrGSEA activity analysis revealed that the gene set of BBR targets was significantly enriched in tumor cell and macrophage clusters. This analysis confirmed at the single-cell level that the focus of BBR’s action is on tumor cells and their surrounding immune accessory cells. In particular, the high activity of hub genes such as SRC and STAT3 in tumor cells and macrophages suggests that BBR may act on both cell types to not only inhibit the malignant behavior of tumor cells but also regulate the function of tumor-associated macrophages, thereby achieving a comprehensive anti-tumor effect.

At the cellular functional level, this study confirmed that BBR effectively inhibits TNBC cell proliferation and colony formation, and significantly induces apoptosis in a concentration- and time-dependent manner. Mechanistic studies revealed that BBR treatment not only downregulated the phosphorylation levels of PI3K, AKT, SRC, and STAT3 proteins but also triggered a marked accumulation of intracellular ROS. Rescue experiments using the antioxidant NAC successfully reversed BBR-induced apoptosis and the inhibition of the PI3K/AKT pathway, further establishing ROS accumulation as an upstream driver in the BBR signaling cascade. Disruption of redox homeostasis may amplify the inhibitory effects of BBR on pro-survival signaling networks by impairing mitochondrial function and activating stress-related kinase pathways (Rijo et al., 2020; Glorieux et al., 2024; Li et al., 2025).

Furthermore, this study is the first to systematically evaluate and confirm the inhibitory effect of BBR on the stem cell characteristics of TNBC. BBR treatment significantly reduced the proportion of CD133^+^ cell subpopulations and downregulated the expression of canonical stemness markers, including ALDH1A and the pluripotency transcription factor SOX2, but also profoundly inhibited tumor sphere formation ability. Cancer stem cells are the source of drug resistance, recurrence, and metastasis (Galassi et al., 2024; Yi et al., 2024; Pan et al., 2025). BBR may fundamentally weaken the long-term recurrence potential of TNBC by inhibiting the SRC/STAT3 pathway, a key pathway that maintains stem cell self-renewal and drug resistance. This finding elevates the antitumor effect of BBR from simple cytotoxicity to targeting the tumor-initiating cells, enhancing its clinical translational value.

Our findings are consistent with previous reports emphasizing the multi-targeted anti-tumor potential of BBR. For instance, several studies have documented BBR’s ability to induce apoptosis in TNBC cells by modulating the PI3K/AKT/mTOR signaling cascade and triggering ROS-mediated oxidative stress (Almatroodi et al., 2022; Zhu et al., 2022), which is strongly supported by our experimental results. However, our study significantly extends the current understanding in two key aspects. First, while earlier research often focused on isolated epithelial mechanisms, our integration of single-cell RNA sequencing revealed that BBR’s primary targets (e.g., SRC and STAT3) are enriched not only in malignant epithelial clusters but also in macrophage populations within the TME. This suggests that BBR’s anti-tumor efficacy may involve a synergistic modulation of both tumor cell intrinsic signaling and the extrinsic immune microenvironment. Second, although BBR has been known to affect breast cancer stemness, we identified the SRC/STAT3 axis as a novel specific regulatory hub for this effect in TNBC. Unlike prior studies that highlighted different pathways such as Hedgehog or Wnt/β-catenin in other subtypes, our structural and functional data pinpoint SRC as a direct binding target of BBR, providing a high-resolution mechanistic link between drug binding and the loss of TNBC stem-like properties.

Although this study has yielded relatively systematic findings, certain limitations still exist. One primary limitation is that the mechanistic investigation was primarily based on *in vitro* cell models; therefore, the anti-tumor activity of BBR *in vivo* and its regulatory effects on the tumor immune microenvironment remain to be further validated in immunocompetent or patient-derived tumor xenograft models. Furthermore, while our core predicted targets (such as SRC, STAT3, and PI3K) were rigorously validated at the protein level via *in vitro* Western blotting, the initial large-scale bioinformatic screening was predominantly based on transcriptomic data due to the current scarcity of large-cohort public proteomic datasets for TNBC. We acknowledge that the lack of global proteomic data represents a limitation, and future research employing mass spectrometry-based global proteomics will be essential to comprehensively map BBR-induced protein interaction networks. Additionally, infiltration analysis and single-cell transcriptomics primarily reflect correlations, and the causal mechanisms remain unclear. While the single-cell RNA sequencing (scRNA-seq) analysis provided valuable exploratory insights, it is constrained by a relatively small sample size *n* = 6 and the lack of an independent validation cohort; thus, these findings should be interpreted as supplementary evidence. Future studies should incorporate conditional gene knockout models and co-culture systems to elucidate the direct or indirect regulatory mechanisms. Lastly, given the low oral bioavailability of BBR, developing novel delivery strategies (such as nanoformulations) to enhance its targeting ability and therapeutic efficacy will be an important direction for promoting its clinical translation.

## 5 Conclusion

By integrating network pharmacology and multi-omics analyses with robust *in vitro* validation, the present study demonstrates that BBR significantly inhibits the proliferation of TNBC cells, induces apoptosis, and attenuates the stemness maintenance capacity of cancer stem cells *in vitro*. Mechanistically, BBR may exert a comprehensive regulatory effect on the malignant biological characteristics of TNBC by modulating the activity of the PI3K/AKT and SRC/STAT3 signaling pathways, accompanied by an elevation in intracellular oxidative stress levels. These findings provide an experimental basis for the potential application of berberine, a multi-target natural compound, in TNBC research, and lay the foundation for the subsequent development of intervention strategies with improved safety profiles.

## Data Availability

The datasets presented in this study can be found in online repositories. The names of the repository/repositories and accession number(s) can be found in the article/Supplementary Material.

## Supplementary material

SUPPLEMENTARY MATERIAL S1 After integrative analysis and removal of duplicate targets, a total of 349 drug- related targets were ultimately identified.

SUPPLEMENTARY MATERIAL S2 A total of 1,049 differentially expressed genes (DEGs) were identified, including 500 upregulated genes and 549 downregulated genes.

SUPPLEMENTARY MATERIAL S3 These 15 feature genes were integrated with 2,830 previously reported TNBC-related genes collected from the GeneCards, OMIM, and TTD databases, resulting in a total of 2,835 TNBC-associated genes.

SUPPLEMENTARY DATA SHEET 1 Supplementary figures.
