# Supplementary material for: Multi-omics integration and experimental validation reveal the mechanism of berberine against triple-negative breast cancer
Source: Front Pharmacol. 2026 May 28;17:1811985. doi: 10.3389/fphar.2026.1811985 (PMC13254460; doi:10.3389/fphar.2026.1811985)
Supplement: Supplementary file 3 [file DataSheet1.pdf]

Supplementary Figures

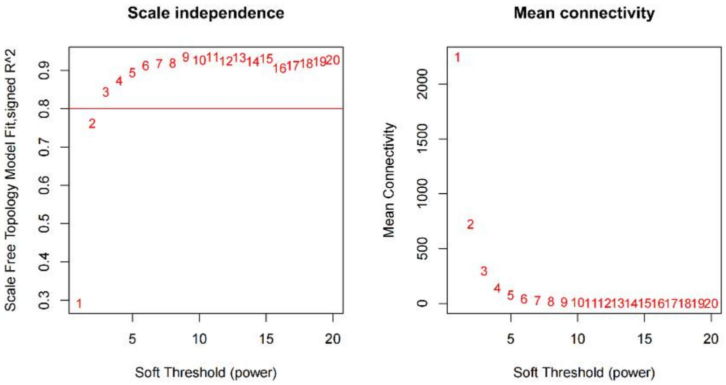

Supplementary Figure 1. Selection of soft thresholds. The soft-thresholding power was set as 3 with a scale-free index greater than 0.8, indicating that connectivity was reasonable.

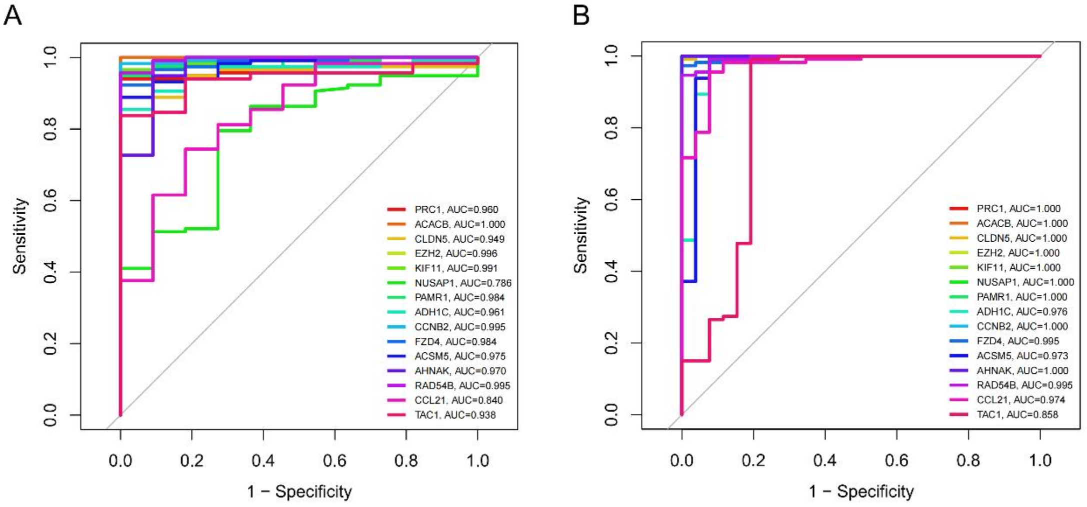

Supplementary Figure 2. (A) ROC curves of the 15 feature genes in the GEO dataset. (B) ROC curves of the 15 feature genes in the TCGA dataset. The AUC values were all greater than 0.8, indicating that these 15 genes possess excellent diagnostic value.

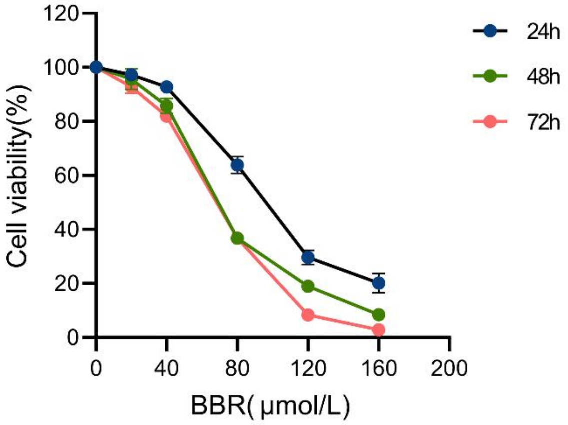

Supplementary Figure 3. Inhibitory effects of BBR on MDA-MB-231 cells in vitro. Cell

viability decreased with increasing BBR concentration. The IC50 values at 24, 48, and 72 h were 94.67, 69.22, and 64.86 μM, respectively.

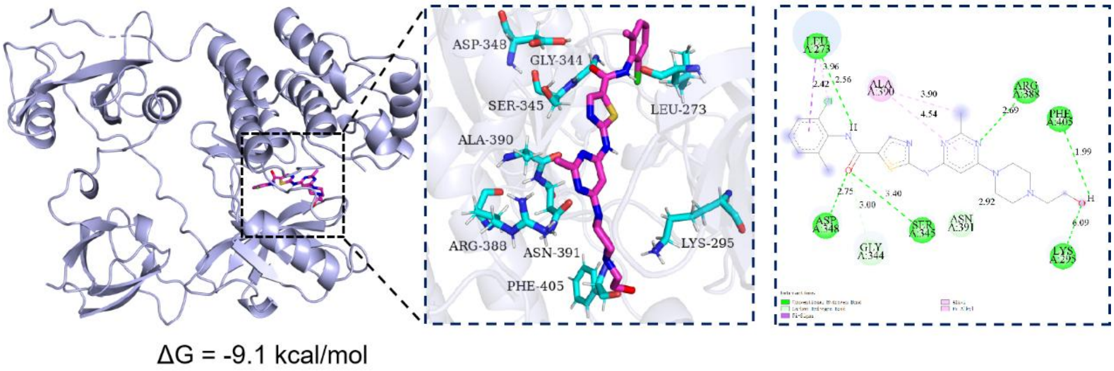

Supplementary Figure 4. Molecular docking simulation of Dasatinib binding to SRC kinase. Left: 3D representation of the SRC kinase domain (cyan cartoon) in complex with Dasatinib (magenta sticks), showing the ligand precisely embedded within the active binding pocket. Center: Close-up view of the binding site illustrating the spatial orientation of Dasatinib relative to key residues (e.g., ASP-348, SER-345, LEU-273).Right: 2D ligand-protein interaction diagram. Green dashed lines indicate conventional hydrogen bonds (labeled with distances in A); pink and purple dashed lines represent hydrophobic interactions (Alkyl/Pi-Alkyl) and Pi-Sigma interactions, respectively. The calculated binding free energy for this pose is ΔG = -9.1 kcal/mol. indicating a high binding affinity between the compound and the target protein.
